# Practice-centred evaluation and the privileging of care in health information technology evaluation

**DOI:** 10.1186/1472-6963-14-243

**Published:** 2014-06-05

**Authors:** Mary Darking, Rachel Anson, Ferdinand Bravo, Julie Davis, Steve Flowers, Emma Gillingham, Lawrence Goldberg, Paul Helliwell, Flis Henwood, Claire Hudson, Simon Latimer, Paul Lowes, Ian Stirling

**Affiliations:** 1 School of Applied Social Science, Faculty of Health, University of Brighton, Mayfield House, Falmer BN1 9PH, UK; 2 Sussex Kidney Unit, Royal Sussex County Hospital, Brighton and Sussex University Hospitals NHS Trust, Eastern Road, Brighton, BN2 5BE, UK; 3 Centre for Innovation Management (CENTRIM), Freeman Centre, University of Brighton, University of Sussex Campus, Falmer BN1 9QE, UK; 4 Clinical Computing Limited, 1 Bath Street, Ipswich IP2 8SD, UK; 5 South Eastern Kidney Patients Association (SEKPA), c/o Sussex Kidney Unit, Royal Sussex County Hospital, Brighton and Sussex University Hospitals NHS Trust, Eastern Road, Brighton BN2 5BE, UK

**Keywords:** Electronic patient records, Telemedicine, Practice, Participatory methods, Care, Evaluation, Capability building

## Abstract

**Background:**

Electronic Patient Records (EPRs) and telemedicine are positioned by policymakers as health information technologies that are integral to achieving improved clinical outcomes and efficiency savings. However, evaluating the extent to which these aims are met poses distinct evaluation challenges, particularly where clinical and cost outcomes form the sole focus of evaluation design. We propose that a practice-centred approach to evaluation - in which those whose day-to-day care practice is altered (or not) by the introduction of new technologies are placed at the centre of evaluation efforts – can complement and in some instances offer advantages over, outcome-centric evaluation models.

**Methods:**

We carried out a regional programme of innovation in renal services where a participative approach was taken to the introduction of new technologies, including: a regional EPR system and a system to support video clinics. An ‘action learning’ approach was taken to procurement, pre-implementation planning, implementation, ongoing development and evaluation. Participants included clinicians, technology specialists, patients and external academic researchers. Whilst undergoing these activities we asked: how can a practice-centred approach be embedded into evaluation of health information technologies?

**Discussion:**

Organising EPR and telemedicine evaluation around predetermined outcome measures alone can be impractical given the complex and contingent nature of such projects. It also limits the extent to which unforeseen outcomes and new capabilities are recognised. Such evaluations often fail to improve understanding of ‘when’ and ‘under what conditions’ technology-enabled service improvements are realised, and crucially, how such innovation improves care.

**Summary:**

Our contribution, drawn from our experience of the case study provided, is a protocol for practice-centred, participative evaluation of technology in the clinical setting that privileges care. In this context ‘practice-centred’ evaluation acts as a scalable, coordinating framework for evaluation that recognises health information technology supported care as an achievement that is contingent and ongoing. We argue that if complex programmes of technology-enabled service innovation are understood in terms of their contribution to patient care and supported by participative, capability-building evaluation methodologies, conditions are created for practitioners and patients to realise the potential of technologies and make substantive contributions to the evidence base underpinning health innovation programmes.

## Background

Evaluation activities can generate important opportunities for reflection, analysis and intervention. The extent to which they do is a question of evaluation design. In the context of health information technologies (IT) used in hospital care, such as electronic record systems and telemedicine, 25 years of research and implementation effort has shown that predicted outcomes prove hard to achieve in practice [1-4]. Conventional positivist approaches to evaluation which measure cost and clinical outcomes alone lack the scope and flexibility to be able to account for and produce potentially valuable ‘lessons learned’ from the unintended and unachieved. Those involved in large scale health IT evaluations have proposed a number of alternative guidelines and approaches to inform research and evaluation [3,5,6].

We add to and build upon this body of work by offering a protocol for health IT evaluation that aligns evaluation with changes achieved ‘in practice’ [7-10] rather than changes that are predicted or envisaged. In line with findings from other significant research on health IT evaluation we argue that a number of recurrent evaluation challenges can be resolved by critically engaging with the questions: *who* is involved in evaluation and *what* are they seeking to achieve? Asking these questions presents an opportunity to focus attention on the priorities and concerns of those taking part in implementing and using health IT. In our case study this led to the direct involvement of patients and clinicians and a specific focus on how health IT could bring improvements in care. It also produced a concern with ‘where’ and ‘when’ health information technology-related changes materialise as changes to the ‘practice of care’ [11,12]. Having collectively established these evaluation priorities and identified them as ‘practice-centred’, we went on to ask the question: how can a practice-centred approach be embedded into evaluation of health information technologies? Our particular interest was in how a practice-centred approach could help shift the focus of health IT evaluation towards improvement in patient care.

We found that aligning evaluation efforts to the articulation of *how* specific capabilities emerged and questions of *who* is in a position to account for the difference those capabilities have made to the practice of care, offered a framework within which to understand, evaluate and communicate the outcomes of implementation efforts. On the basis of our experience, we make the case that those ‘best placed’ to comment on and assess technology-related change or lack thereof, are those involved in the work of caring - or ‘care practice’ as we refer to it - and include healthcare staff, patients and informal carers. We therefore propose that a participatory model of implementation supported by action learning and a programme of collaborative, participatory research can positively influence the outcomes of health information technology implementation and ongoing use. Our contribution, drawn from our experience and the case study provided, is a protocol for practice-centred, participative evaluation of technology in the clinical setting.

### The case study: the sussex renal innovation programme (SRIP)

The Sussex Renal Innovation Programme (SRIP) is a regional programme of technology-enabled innovation in renal services (2009 - ongoing) [13]. The programme was underpinned by the procurement and implementation of a regional EPR system that would support, in addition to many other clinical activities, new telemedicine and teleconferencing capabilities. Medical care for kidney disease is typically organised around a specialist unit (which may or may not include a kidney transplant centre), satellite dialysis units and home dialysis support. The information intensive nature of renal care coupled with a need to provide services across a geographically dispersed population enhanced the need for a ‘whole system’ approach. Additionally, a significant characteristic of the kidney patient community is that the capacity exists for them to be able to access information from their electronic patient record via an open source technology originating from the renal community called Renal Patient View [14]. The new EPR system would enable all patients in this region to have access to this facility.

A distinctive element of the SRIP was its stated aim and consequent adherence to a ‘user-centred’ [15] approach which led to the constitution, very early on, of a multi-disciplinary, patient-involved action learning group called the ‘SRIP Action Learning Group’ (ALG) [16]. Membership of the ALG was by invitation of the clinical lead nephrology consultant for the SRIP. A typical meeting would include a Ward Nurse Manager, a Nurse Specialist, the Renal Data Manager, an Administration Manager, the Haemodialysis Unit Manager, a Change Facilitator, a Practice Educator, a Social Care expert, a junior doctor on the renal unit and a Patient Representative [17].

The ALG began meeting during the strategic development of the programme and these discussions informed the EPR procurement process. The EPR procurement process was carried out participatively with all ALG members invited to attend product demonstrations and engage in discussions with potential suppliers. A specialist, renal EPR system was purchased and in addition, a data analytics module was commissioned along with two ‘integration pieces’: one that would feed results from regional microbiology and virology laboratories into the EPR; and one that would link the EPR to the patient-facing portal Renal Patient View. The video clinics and teleconferencing component was initially progressed as a pilot project by a small sub-group of the ALG working in collaboration with a local, university-based innovation centre who provided the necessary technical equipment and expertise [18].

## Methods: developing a participative, practice-centred approach

From its inception, the ALG invited ‘outsider’ researchers and health policy specialists to group meetings in order to bring alternative perspectives to discussions. A research team from a local university was invited to join the programme on a 30 day consultancy contract to be carried out over a 6 month period between April 2010 and October 2010. It was assumed that this time period would include pre-implementation planning, implementation itself and allow one month for post-implementation observation. Having stated their intention to be ‘partners in innovation’, representatives of the EPR supplier also became active members of the ALG attending meetings from April 2010.

EPR and telemedicine implementations are widely acknowledged as being complex and contingent areas of technology-enabled service innovation [5,6]. Whilst broad outcome measures for the SRIP were defined in advance the dynamic and evolving character of events meant specific benefits were hard to pinpoint due to the intensive product development, technology infrastructure work, hardware procurement and implementation activities taking place [6].

Inevitably, the EPR implementation over-ran and ‘go live’ was delayed from September 2010 to December 2010. This altered the original implementation evaluation design which was scheduled to finish after 6 months. By mutual agreement the evaluation period was extended so that the university team could see what changes to practice the EPR implementation effort would finally produce. Ongoing ALG meetings, a super user strategy and cascade training model ensured that clinical staff were prepared for ‘go live’ and that adequate support would be available ‘on the shop floor’.

As is often the case, the integration aspects of the programme took longer than expected and so benefits associated with Renal Patient View and the integration of laboratory results were delayed. Similarly, ‘go live’ activities focused on the main unit in the first instance with satellite units scheduled to follow at a later date. Within the main unit the decision had been taken that the ward would not go live at this point due to concerns that it would lead to ‘dual administrative systems’ (i.e. paper and electronic) which ward nurses contended would impact on time available for direct patient care [19]. Nonetheless, for the remaining areas of the renal unit the clinical team, technology supplier, data manager and administrative staff achieved an impressive ‘business as usual’ switch to the new system (apart from an issue affecting the production of patient letters for which a workaround had to be found).

Initially, in the weeks following ‘go live’ it appeared that ‘nothing had changed’. In many respects this was an indication that the implementation had been a success, but in other respects it was disappointing not to see immediate benefits. Process mapping in the months preceding implementation had identified specific opportunities for achieving service efficiencies through use of the EPR. However, it was clear that these planned improvements would require significant service innovation effort on the part of staff and would take up a substantial amount of time and effort. As those involved in the SRIP were already contributing a considerable amount of time to implementation activities, which in turn were taking longer than predicted, the timescale for these planned improvements had to move from pre-implementation to post-implementation. However, unplanned improvements did occur fairly soon after implementation. For example, on seeing how the system worked *in situ*, an ALG member and nurse manager identified a means through which the new system could support ‘named nurse meetings’ and was able to act on this almost immediately. Through encountering these examples ALG members became conscious that the benefits of the system would not ‘just happen’ post-implementation but would have to be pushed through by nursing and medical staff.

From this point, it was nursing staff who progressed the ongoing development of the EPR system and post-implementation benefits realisation, with focussed, strategic input from the lead consultant. Although another consultant and a hospital registrar would attend ALG meetings occasionally, the group was Chaired and predominantly attended by nurses. Nurses took on leadership roles in key parts of the programme delivery such as training, technical support and hardware procurement/trialling [20]. They became very knowledgeable about the EPR product, issues that affected system use and data quality, and how to address these issues through negotiation with the supplier [21]. They instituted processes for: tracking ‘change requests’ and ‘bug fixes’; held the supplier to account for slippages in the implementation timeframe; and insisted that the product developers responded to needs arising from opportunities they identified for EPR-supported service improvement. Involvement of supplier representatives as members of the ALG and the capacity this created for direct, face-to-face interaction, enhanced supplier responsiveness, encouraging clinician engagement.

The EPR implementation occupied so much staff time that video clinics were not piloted until 8 months after the EPR went live. Two clinics with 4 patients in each were successfully piloted [18]. The hardware used for the pilot did not belong to the Trust and so a procurement process was entered into with local trust information services. Procurement and subsequent trialling processes were still ongoing as of March 2013.

The external evaluation team, originally contracted to evaluate the EPR system implementation, remained engaged during this post-implementation period with one member now acting as a participant researcher within the ALG. With numerous post-implementation issues to resolve it became clear that knowing ‘when’ the system would bring a change to practice and who within the broader renal clinical team would enact that change would be vital [22,23]. A ‘watch list’ of benefits that were ‘on the horizon but not here yet’ was constructed. It was difficult to attach precise timeframes to ‘when’ benefits would be realised as work was still very much ongoing. Four years on from implementation several key pieces of the original implementation plan remained uncompleted, such as the integration of blood test results from regional laboratories with the EPR. Significant efficiencies and patient benefits could be realised from this element of the programme. However, the multi-stakeholder nature of this element and the need for input from institutional level IT departments has resulted in slow progress.

## Key findings

It is well-documented that programmes of innovation in service delivery that involve telemedicine and EPR system implementation present distinct evaluation challenges due to their typically dynamic and evolving nature [24-26,5,6]. Our experience of the SRIP supports these findings. However, whilst the programme did not proceed according to pre-specified timescales and outcome measures, the participative and practice-centred approach to evaluation enabled alternative guiding principles to emerge. These principles were unified by our co-participants’ over-riding concern with care and how it is practiced. We embedded this concern in our approach and refer to it as ‘privileging care’. We discuss these guiding principles and the privileging of care below before going on to show how they can form the basis of a protocol for future capability building evaluation research.

### Competing demands and the defence of ‘time to care’

**Figure 1:**
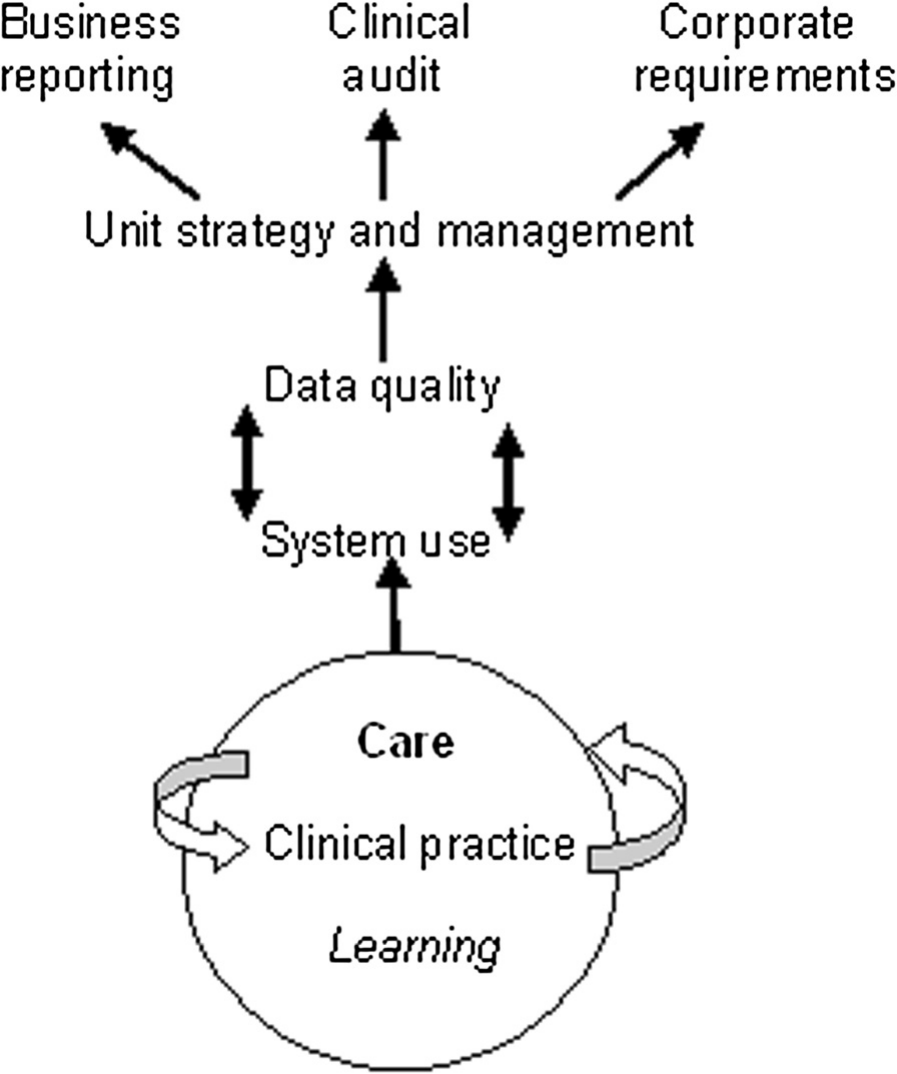
Existing data flow model.

Clinical practitioners had limited time to commit to evaluation activities. This response was partly due to a broader climate of perceived ‘over-audit’ and partly due to the significant time that implementation activities were taking up [27]. Clinical staff were actively engaged in collecting and collating a range of information to meet business, clinical and corporate requirements. From their perspective this data was ‘sent out’ of the unit and where results were returned they formed part of annual or quarterly feedback cycles that did not improve the information held on patients for whom they were currently responsible [28]. Clinicians with management and leadership responsibilities expressed similar concerns regarding a lack of information to reflect the immediate, day-to-day organisation of services. This state of affairs is represented in Figure 1 as a simple data flow model. In this model, clinical practice, care and learning are regarded as fundamental activities that are nonetheless perceived as ‘one step removed’ from day-to-day system use and data quality. In this model the purpose of the EPR system is understood to be more closely allied to business and cost reporting, clinical audit and corporate, Trust-wide requirements than care, clinical practice and learning.

ALG members were of the unanimous opinion that the EPR system had to lessen rather than add to the audit and administration burden, releasing time for direct patient care, or else it would be unusable. This was particularly pertinent in the case of ward nursing in the main renal unit [19]. There was also consensus that the system should support ‘better care’ and ‘learning’ about what ‘helped patients’ first and foremost, and that other reporting demands should be served as a consequence of meeting those aims.

### Privileging care

**Figure 2:**
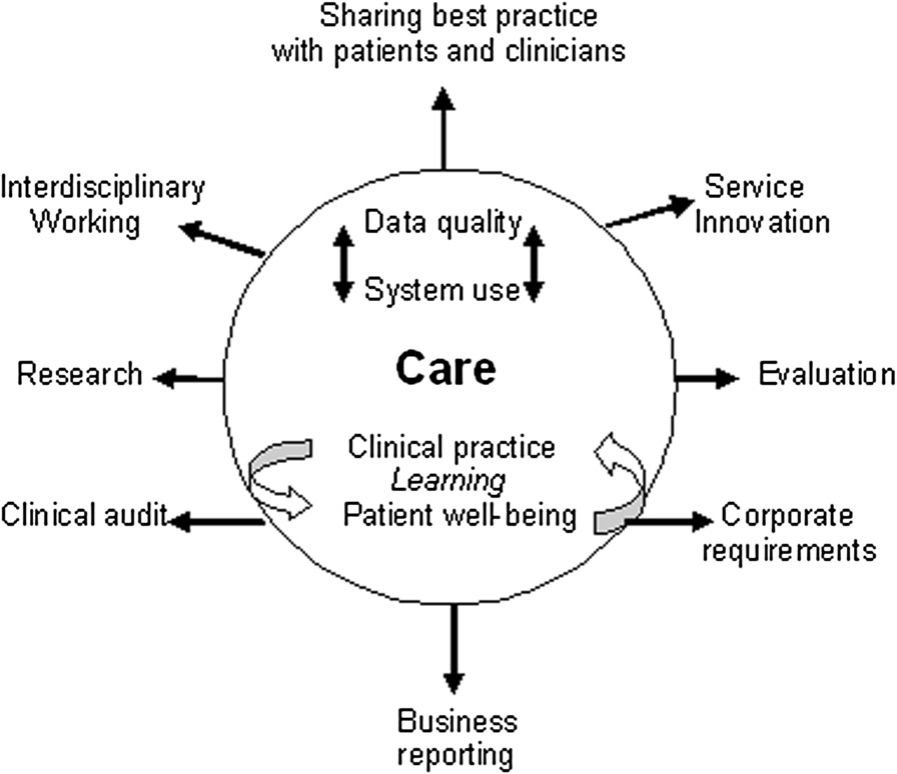
Ideal model of data generation and use.

The priorities expressed by ALG members concerning what they hoped to achieve from the SRIP guided implementation planning and informed the emerging framework for evaluation. ‘Privileging care’ described an aspirational position whereby new technology would enhance care by creating opportunities to reflect upon and improve clinical practice, and support patient well-being. In an ideal scenario, system use and data quality would relate directly to these goals and other data needs met as a consequence of serving these ends. In this sense, other needs were not only those related to clinical audit, business reporting and corporate requirements for information, but also greater recognition of the interdisciplinary use of data across the clinical team. Sharing best practices and insights gained into patient care through use of the system in itself becomes a source of learning and of new ideas for service innovation. With improvements in data quality, EPR data also become available for use in staff research projects encouraging a culture of research around health IT use that supports ongoing development. Figure 2 describes this position in terms of an ideal model for data generation and use.

### Patient involvement

Patient involvement in the ALG altered the way in which care and evaluation were conceived of and discussed. Developing an ongoing model of evaluation that assumed the involvement of patients brought about a redefinition of what kinds of care were significant in the context of the EPR. Having the patient representative participating in the ALG broadened the notion of what constitutes renal care and who is involved in that care. Conventionally, within healthcare contexts, care is defined in terms of medical and clinical practice, i.e. professional care-*giving*. These constructions can conceal the work that patients and informal carers put into coping with illness and potentially obscure the ways in which patients are active in their own ‘self’ care [29-31]. In the development of the evaluation methodology this provoked a shift from understanding practice as alluding specifically to ‘medical’ or ‘clinical practice’ to ‘care practice’ defined as knowledgeable activities engaged in as part of everyday routines associated with both formal and more informal forms of care [11,19,32].

### Carer roles

The kidney patient representative routinely emphasised the wider network of care and, in particular, the significance of carer involvement in patient care [32,33]. For renal patients, the involvement of family members, friends or other representatives can be crucial, in some cases determining whether or not a patient can consider home dialysis options. For patients’ carers, having (via patient consent) access to information that can support informal care activities offers significant benefits. A NHS Kidney Care evaluation of Renal Patient View [13] analysed usage statistics which showed that use of Renal Patient View increased when kidney patients experienced acute episodes and were admitted as in-patients. Given that many hospital wards do not offer wireless internet access and that most inpatients have immediate access to their blood results via their nurse or consultant, the conclusion drawn by the evaluation was that it was carers logging onto the system from home for updates that caused a spike in usage statistics.

### Invisibility of systems to stakeholders

As with most care record systems, it is healthcare professionals rather than patients who are typically understood as ‘users’ of the system. Patients are likely to never see, nor will they ever directly input, data into their hospital’s EPR system, yet that system will form a cornerstone of their care. This creates a potential barrier to patient and public understanding of ethical considerations and potential safety issues that might arise from EPR and biomedical data use [34,35]. In terms of patient EPR awareness, this situation is slightly different for renal patients living in areas where Renal Patient View is available. In these cases, all patients, carers and GPs have the opportunity to see a view of the EPR data and therefore act on the basis of new test results [13]. For the patient representative in our example, this made community engagement in the implications and outcomes of the EPR implementation more compelling and tangible, translating a complicated and remote institutional IT change into an issue of immediate relevance to patients and questions of care [36]. It also altered the evaluation approach by increasing the scope for involving patients in evaluation data collection efforts and crucially, in evaluation design.

This model expresses a desire for a direct and positive association between system use and patient care, based on a timely feedback cycle where patient data is central to ongoing processes of learning, service improvement and patient education. The antithesis of this model – that EPR system use would increase time spent on data entry and administration without releasing time to care – was positioned by participants as not just undesirable but untenable. The question of whether EPR system use would lessen administration and create more opportunities to review patient information in real time, remained a central concern throughout the implementation and ongoing development process [19].

### Implications: a protocol for participative, practice-centred evaluation that privileges care

The external researchers involved in coordinating evaluation efforts identified generalizable principles from the case study described above. These principles were informed theoretically by the notion of ‘practice’, which places *a priori* emphasis on what it is that people do, from day-to-day, in specific contexts and settings. A practice-centred approach to evaluation is supported by a participative approach to evaluation design, data collection and analysis because one of its primary aims is to identify and engage those people ‘best placed’ to see ‘when’ and ‘how’ technology-related changes materialise. This process is emergent and ongoing because it is accepted at the outset that the outcomes of health IT implementations are cannot be fully known in advance. Taking this position has allowed us to identify and evaluate examples of: changes to practice that had been predicted; changes to practice that had been predicted but which did not proceed as expected; changes to practice that did not materialise; and changes that occurred as the result of unanticipated, innovative interactions between the system and those seeking ways to incorporate it into their care practice.

Table 1 provides practical guidelines for designing and undertaking a practice-centred evaluation. Our approach incorporates both practice-centred and participative elements and so the table below provides guidelines that refer to both elements respectively. Whilst a practice-centred evaluation does not necessarily require a participative approach to evaluation activities, we found that the two were very closely linked. Therefore, in the table below, the left-hand column describes how to design evaluation activities so that they are practice-centred and in this case, privilege care. In addition, the right-hand column highlights ways to support participative evaluation work and promote critical reflection on the nature of and extent to which participation is achieved.

**Table 1:** A protocol for practice-centred evaluation that privileges care

**Practice-centred**	**Participative**
- Organise evaluation around care ‘scenarios’ where EPR related change has (or has not) occurred	- Organise evaluation activities around core values and priorities of those participating. In our case: patient wellbeing, learning and care
- Expect opportunistic or emergent change to be generative of significant benefits and problems	- Encourage those experiencing or producing health information technology-related changes to participate in designing and where possible carrying out evaluation of those changes
	- Work collaboratively in groups including at least one person who is directly involved in the change being evaluated
	- Use multiple data sources (e.g. questionnaire data, system usage data, interviews), admit diversity of opinion and resist synthesis or judgement
	- Encourage collective reflection on the boundary of involvement in evaluation (i.e. ‘who’ or ‘what’ is included/excluded) and seek ways to counter persistent exclusion
- Develop an evaluation ‘watch list’ of areas of practice where it is anticipated EPR related changes to practice will occur in order to capture *if*/*when* those changes occur	- Report findings in a way(s) that ‘speaks’ to the community of practice concerned
- Include in this list: change anticipated as part of specific service improvement activities; and/or change relating to EPR functionalities that have been either speculated upon and/or specified in advance; and/or potential negative changes to practice	
- Capture ‘before change’ data relevant to both practice and outcomes, where possible or relevant	
- Wait for change to materialise ‘in practice’ before collecting ‘after change’ data	
- Actively include care scenarios where EPR related changes have: not proved possible; only partially been achieved; required an unanticipated amount of effort; proven exceptionally slow to achieve; or proven unachievable	

Using this approach, SRIP evaluators have so far conducted evaluations using: ‘before’ and ‘after’ evaluation data; case study based description; and patient questionnaires [37,38,18]. They have reported their findings at conferences attended by clinical colleagues, nurse research seminars within the hospital trust, at a research symposium to designed to stimulate interest from local universities in secondary care informatics and at a national user group meeting they coordinated in collaboration with their EPR supplier.

### Future research agenda

We have argued the case for a participative, practice-centred approach to health information technology evaluation that privileges care. We have done so on the basis that such an approach supports the production of evidence pertaining to *how*, *when* and *for whom* health information technology systems can be said to change care and care practice. Based on our experience, we argue that patient involvement and nurse leadership is likely to be at the forefront of efforts to realise health information technology benefits and ensure these benefits are oriented toward better care for patients. Supporting nurses in this role will require capability building in research and evaluation, which in turn requires the recognition and endorsement of these skills in programmes of education and professional development. There is potential for participative, action learning-based evaluation efforts to support the development of health IT knowledge within and across hospital trusts. We argue that this does not just support shared understanding but also the ‘in practice’ realisation of health IT and its potential. This particular finding holds implications for health IT producers and hospital Trust strategic leads as much as it does patients, carers, clinicians and the general public. From our experience, there is clear enthusiasm for sharing experience that can and should be mobilised. Four years after ‘go live’, the ALG is still meeting and is keen to share what they have learned with clinical groups participating in the hospital-wide EPR implementation taking place in their hospital trust and other users of renal EPR systems.

## Competing interests

The authors declare that are no competing interests.

## Authors’ contributions

The ideas presented here emerged from collaborative discussions held in the SRIP Action Learning Group (ALG). All authors were members of the ALG apart from SF and FH who (along with MD) formed the university evaluation team. Early versions of the text were presented as summative feedback to the ALG where the text, ideas and arguments were commented on extensively. RA, FB, SF, EG, LG, PH, FH, CH, PL and IS all contributed to several revisions of the manuscript and the intellectual development of the article. All authors read and approved the final manuscript.

## Pre-publication history

The pre-publication history for this paper can be accessed here:

http://www.biomedcentral.com/1472-6963/14/243/prepub
